# Induction of Anti-Tumor Immune Responses by Peptide Receptor Radionuclide Therapy with ^177^Lu-DOTATATE in a Murine Model of a Human Neuroendocrine Tumor

**DOI:** 10.3390/diagnostics3040344

**Published:** 2013-10-02

**Authors:** Yin Wu, Andreas Klaus Pfeifer, Rebecca Myschetzky, Rajendra Singh Garbyal, Palle Rasmussen, Ulrich Knigge, Michael Bzorek, Michael Holmsgaard Kristensen, Andreas Kjaer

**Affiliations:** 1 Peter Gorer Department of Immunobiology, Guy’s Hospital, Great Maze Pond, London, SE1 9RT, UK; 2 Cluster for Molecular Imaging, Faculty of Health Sciences, University of Copenhagen, Copenhagen, 2200, Denmark; E-Mails: andreas.pfeifer@mac.com (A.K.P.); rebeccam@mfi.ku.dk (R.M.); ulrich.peter.knigge@regionh.dk (U.K); akjaer@sund.ku.dk (A.K.); 3 Department of Clinical Physiology, Nuclear Medicine and PET, Rigshospitalet, Blegdamsvej 9, Copenhagen, 2100, Denmark; 4 Department of Clinical Pathology, Næstved Hospital, Ringstedgade 61, Naestved, 4700, Denmark;E-Mails: raje@regionsjaelland.dk (R.S.G.); mibz@regionsjaelland.dk (M.B.); mick@regionsjaelland.dk (M.H.K.); 5 Risø National Laboratory, Danish Technical University, Anker Engelunds Vej 1, Lyngby, 2100, Denmark; E-Mail: pall@dtu.dk; 6 Department of Surgery C, Rigshospitalet, Blegdamsvej 9, Copenhagen, 2100, Denmark

**Keywords:** PRRT, ^177^Lu-DOTATATE, CD86+, CD49b+, FasL+, dendritic cells, NK cells

## Abstract

Peptide receptor radionuclide therapy (PRRT) is a relatively new mode of internally targeted radiotherapy currently in clinical trials. In PRRT, ionizing radioisotopes conjugated to somatostatin analogues are targeted to neuroendocrine tumors (NETs) via somatostatin receptors. Despite promising clinical results, very little is known about the mechanism of tumor control. By using NCI-H727 cells in an *in vivo* murine xenograft model of human NETs, we showed that ^177^Lu-DOTATATE PRRT led to increased infiltration of CD86+ antigen presenting cells into tumor tissue. We also found that following treatment with PRRT, there was significantly increased tumor infiltration by CD49b+/FasL+ NK cells potentially capable of tumor killing. Further investigation into the immunomodulatory effects of PRRT will be essential in improving treatment efficacy.

## 1. Introduction

Neuroendocrine tumors (NETs) are a heterogeneous group of epithelial neoplasms of both neurological and endocrine differentiation and are characterized by their common expression of chromogranin A and synaptophysin [1]. NETs are found mostly in the gastrointestinal tract and bronchopulmonary system, though they have also been described in many other organ systems [2,3]. Most NETs are well differentiated displaying a low mitotic and proliferative status associated with slow growth patterns compared to other malignant carcinomas [4]. Despite their slow growth, the variable anatomical sites from which NETs arise and their difficult diagnosis contributes to a late clinical presentation, often following metastatic spread [5]. 

Current treatment of metastatic NETs remains centered on disease control. The historical median survival was around 2 years but with aggressive multimodal treatment by surgery, radiotherapy and peptide receptor therapy this has now been extended to 8 years [4]. The lack of a curative treatment in advanced disease and an increasing incidence make these tumors clinically relevant and also highlight the need for research into novel therapies.

Peptide receptor radionuclide therapy (PRRT), a recently established form of systemic targeted radiotherapy, has been used with success in improving median survival times for patients with advanced metastatic NETs [4,6,7,8,9]. Although NETs are a highly diverse and heterogeneous group of cancers, large subsets express high levels of somatostatin receptors (SSTR) [10]. This high expression has led to the development of synthetic somatostatin analogues conjugated to radioisotopes like ^111^Indium (^111^In) for use as tracers in the imaging of NETs and metastases [11]. The success of these imaging techniques to selectively target SSTR expressing tumor cells led to the development of targeted therapeutic delivery of ionizing radiation to treat NETs. One current treatment regime uses the radioisotope ^177^Lutetium (^177^Lu) conjugated to the somatostatin analogue [DOTA,D-Phe^1^,Tyr^3^]-octreotate (^177^Lu-DOTATATE) [6,7,12]. ^177^Lu is a combined beta and gamma emitter. The high energy and short range ionizing beta radiation from ^177^Lu is presumed to be the mechanism by which tumor regression and control is achieved. Despite promising results in a limited number of clinical trials, there is still very little evidence in the literature on the mechanism by which tumor control is achieved. 

It is well documented that anti-tumor immune responses, particularly in the form of CD8+ cytotoxic T lymphocytes (CTL) and cytotoxic natural killer (NK) cells, are important in the control and eradication of tumor tissue [13,14,15]. Moreover, there is now evidence that radiotherapy itself induces specific anti-tumor responses able to limit tumor growth and in some cases eradicate tumor tissue independent of the direct effects of radiation [15]. Radiotherapy induced damage may lead to the release of tumor antigens which are then processed and presented to the immune system to activate an anti-tumor response.

The aim of the present study was therefore to investigate the ability of ^177^Lu-DOTATATE PRRT therapy to induce an anti-tumor immune response in an *in vivo* xenograft model of human NET. 

## 2. Materials and Methods

### 2.1. Tumors and Mice

All animal studies were conducted in compliance with Danish laws governing animal experimentation. Female nude NMRI mice were purchased from Taconic Europe (Lille Skensved, Denmark) and maintained for two weeks in the animal facilities in the Department of Experimental Medicine, University of Copenhagen, Denmark. NCI-H727 cells (ATCC, Manassas, VA, USA), a cell line derived from a well-differentiated human bronchial NET, were cultured in RPMI medium 1640+ GlutaMAX (Invitrogen, Carlsbad, CA, USA) supplemented with 10% fetal bovine serum (Biological Industries, Israel) and 1% penicillin-streptomycin (Invitrogen) in a humidified atmosphere of 5% CO_2_ at 37 °C in our cell laboratory. At 8 weeks old the mice received subcutaneous injections of 1 × 10^7^ NCI-H727 cells suspended in 1 × 100 uL RPMI medium mixed with 100 mL Matrixgel^TM^ Basement Membrane Matrix (BD Biosciences, San Jose, CA, USA) into either upper flank. Engrafted cells were allowed to establish and grow for two weeks to maximum size of 1 cm in diameter. T cell deficient nude mice were chosen over inmmunocompetent mice to avoid T cell mediated rejection of the xenografts. 

### 2.2. ^177^Lu-DOTATATE Injection and Tumor Preparation

Mice were divided into treatment and control groups. Treatment mice were injected with a high target dose of 30–40 MBq or a low target dose of 3–4 MBq of ^177^Lu-DOTATATE in 200 µL of sterile physiological saline through the tail vein. Control mice were left untreated. Activity was measured on a Amersham ARC-120 dose calibrator (Capintec, NJ, USA) and estimated delivered dose calculated as the decay-corrected difference between initial counts present in the injection syringe and residual counts in injection. Mice were kept alive for 3 days before sacrifice and dissection of tumors to coincide with peak acute inflammatory responses. After dissection, tumors were weighed and activity counted in a Perkin Elmer Lifescience 2480 Wizzard automatic gamma counter (Waltham, MA, USA). For immunohistochemistry (IHC), tumors were snap frozen in tissue freezing medium. For flow cytometry, tumors were cut into small pieces and soaked in trypsin/EDTA at 4 °C overnight.

### 2.3. Immunohistochemistry

Immunohistochemical studies were performed on frozen tumor sections. Briefly, frozen sections (5 μM) were fixed in 10% buffer formalin for 10 min before immunostaining and placed in 0.05 M Tris-buffered saline (TBS). Endogenous peroxidase activity was quenched with 0.5% H_2_O_2_ in 0.5% sodium azide/TBS for 20 min, followed by a wash in TBS. The slides were incubated with primary anti-mouse CD86 (B7-2) FITC (Clone GL1) (eBioscience, San Diego, CA, USA) diluted 1:100 in Power Block solution (BioGenex, Fremont, CA, USA) for 30 min at room temperature (RT). Sections were the washed in TBS (3 × 5 minutes) and incubated with rabbit anti-FITC 1:250 (Invitrogen) for 20 min at RT. After a further 3 washes in TBS, reactions were detected with Super Sensitive polymer-HRP reagent (Biogenex, Fremont, CA, USA) and visualized with ImmPACT DAB substrate chromogen solution (Vector Laboratories, Peterborough, UK) following the manufacturer’s instructions. Negative controls were performed by omission of the primary antibody. Finally, slides were rinsed in water, counterstained with Mayer’s hematoxylin and coverslipped. 

### 2.4. Flow Cytometry

After overnight incubation at 4 °C in trypsin/EDTA, samples were incubated at 37 °C for 30 min to allow for tissue dissociation and single cell suspension. Cells were counted on a hemocytometer and up to 10^6^ cells were seeded in 96 well plates for further immunostaining. Cells were Fc blocked in 10% heat inactivated fetal calf serum (FCS) in FACS buffer (1% BSA in PBS) for 30 min at 4 °C to prevent non-specific staining. Cells were stained with anti-human Fas(CD95)-PE-Cy5 (BD Pharmingen), anti-murine CD49b-APC, anti-murine CD86-PE-Cy5 and anti-murine FasL(CD-178)-PE (eBioscience, San Diego, CA, USA) according to manufacturers’ instructions for 30 min at 4 °C. Cells were washed twice in 200 µL of FACS buffer before fixing in 4% formaldehyde in PBS. Flow cytometry was performed on a BD FACSCalibur and analyzed on FlowJo (TreeStar).

### 2.5. Statistical Analysis

Statistical analysis of the data was performed using the statistical software SPSS, version 17.0 (SPSS, Chicago, IL, USA). Linear regression was used to analyze dose-dependent CD86+ cell responses while student´s t-tests were applied for the analysis of the remaining data. A two-sided *p* < 0.05 was considered statistically significant. 

## 3. Results

### 3.1. Delivery and Actual Tumor Uptake of ^177^Lu-DOTATATE

**Figure 1:**
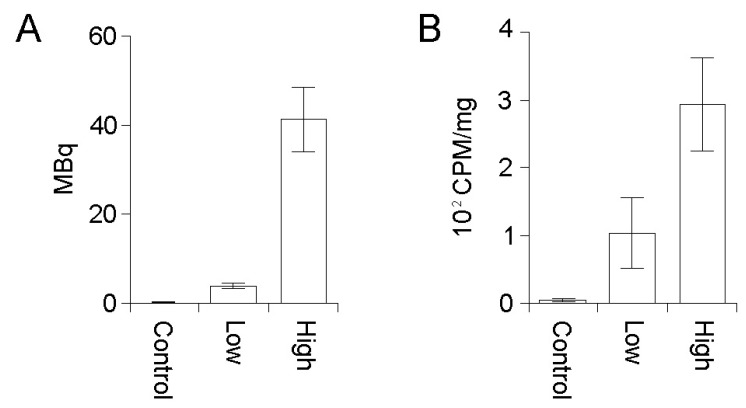
(**A**) Representative experiment showing total injected dose (MBq) of ^177^Lu-DOTATATE injected into each animal was calculated by the difference between the initial activity drawn up into each syringe and the residual activity after injection into the tail vein. Control mice received no ^177^Lu-DOTATATE. Control n = 4. Low dose n = 6. High dose n = 6. The mean ± S.D. is plotted. Representative experiment group is shown. (**B**) Excised tumors were weighed and absorbed dose determined in a gamma well counter. Relative activity absorbed is expressed as counts per minute per milligram of tissue (cpm/mg). Control n = 8. Low dose n = 12. High dose n = 12. The mean ± S.D. is plotted. Representative experiment groups are shown.

Total delivered doses of PRRT were calculated as the decay corrected difference between the residual and initial doses drawn up in each injection syringe (Figure 1(A)). Mice in the high dose treatment group received a mean of 41.2 MBq (±7.2 MBq) while mice in the low dose group received a mean of 3.84 MBq (±0.36 Mbq) of ^177^Lu-DOTATATE (Figure 1(A)). Despite an average ten-fold difference in total delivered dose between the high and low dose treatment groups, we consistently found only an average three-fold difference in actual tumor delivery of ^177^Lu-DOTATATE (Figure 1(B)).

### 3.2. ^177^Lu-DOTATATE Treatment Increases Tumor Infiltration by CD86+ Antigen Presenting Cells

**Figure 2:**
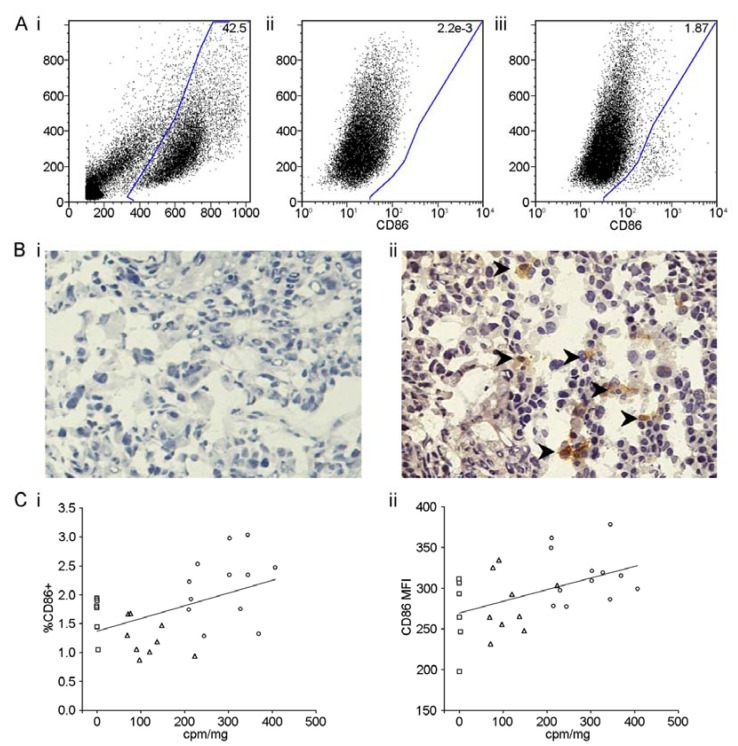
(A-i) Representative flow cytometric dot plot to show gating by forward (x-axis) and side scatter (y-axis) on engrafted tumor and murine immune cell populations with percentage of cells included within gate shown in the top right. (A-ii) Representative plot of unstained tumor preparations (side scatter y-axis, CD86 x-axis) used to define CD86 positive and negative populations. Percentage of CD86+ cells (defined as those included within the gate) shown in the top right. (A-iii) Representative plot of tumor preparations stained with anti-murine CD86. Percentage of CD86+ cells shown in the top right. (B-i) Representative negative control immunohistochemical staining of frozen section preparations of tumor tissue. (B-ii) Representative immunohistochemical staining of CD86+ cells in frozen section preparations of tumor tissue. (C-i) The percentage of CD86+ cells, as gated for in Figure 2(A-i), plotted as a linear regression against absorbed dose of ^177^Lu-DOTATATE (cpm/mg) shows a significant correlation between ^177^Lu-DOTATATE dose and increasing infiltration of CD86+ murine dendritic cells (DCs). R = 0.483, *p* = 0.011. (C-ii) The median fluorescence intensity of CD86 of cells within the CD86+ gate shown in Figure 2(A-i) plotted as a linear regression against absorbed dose of ^177^Lu-DOTATATE (cpm/mg) shows a significant correlation between ^177^Lu-DOTATATE dose and increasing intensity of CD86 expression on murine DCs. R = 0.468, *p* = 0.014.

To assay the effect of ^177^Lu-DOTATATE treatment on APC tumor infiltration and capacity to present tumor antigens, we used flow cytometry and immunohistochemistry to look for CD86 expressing cells in tumor preparations. For flow cytometry, cells were gated on forward and side scatter (Figure 2(A-i)). Cells were blocked with 10% FCS to prevent non-specific staining and unstained samples were used to control for auto-fluorescence and to define CD86 negative cell populations (Figure 2(A-ii)). A small population of CD86+ cells could clearly be identified by flow cytometry within tumors (Figure 2(A-iii)). Immunohistochemical stains of frozen tumor sections directly confirmed infiltration of CD86+ cells into tumor tissue (Figure 2(B-i) and Figure 2(B-ii)).

CD86+ cells could be found infiltrating tumors even in the absence of ^177^Lu-DOTATATE (Figure 2(C-i) and Figure 2(C-ii)) most likely reflecting some degree of inflammation at the site of graft and expanding tumor. Treatment with increasing doses ^177^Lu-DOTATATE correlated with both significantly increased percentages of infiltrating CD86+ cells (R = 0.483, *p* = 0.011) and increased CD86 intensity (R = 0.468, *p* = 0.014) (Figure 2(C-i) and Figure 2(C-ii)).

### 3.3. ^177^Lu-DOTATATE Treatment Increases Tumor Infiltration by Activated CD49b+/FasL+ NK Cells

**Figure 3:**
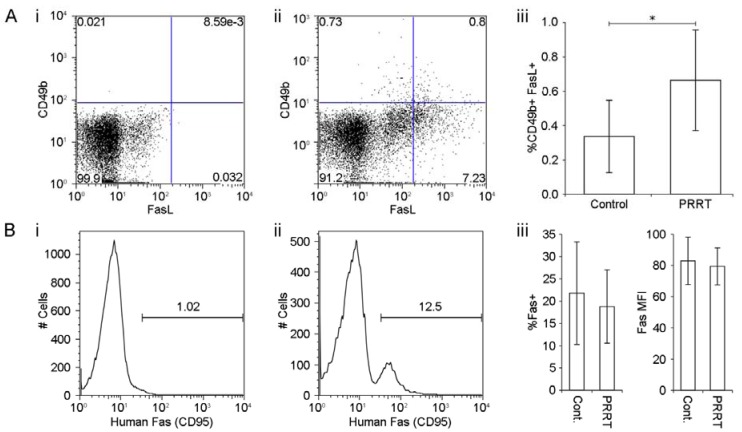
(**A-i**) Representative flow cytometric dot plot of unstained tumor preparations used to define CD49b and FasL (CD178/CD95L) positive and negative populations. Percentage of FasL+/CD49b+ NK cells (defined as those included within the gate) shown in the top right. (**A-ii**) Representative plot of tumor preparations stained with CD49b and FasL. Percentage of double positive FasL+/CD49b+ NK cells shown in the top right. (**A-iii**) There was a significantly increased infiltration of FasL+/CD49b+ NK cells in tumors after treatment with low dose ^177^Lu-DOTATATE. The mean ± SD of 8 control tumors and 12 treated tumors is plotted. *p = 0.0143 unpaired, two-tailed T-test. (**B-i**) Representative plot of unstained tumor preparations used to define human Fas (CD95) positive and negative populations. Percentage of Fas+ cells (defined as those included within the gate) shown in the top right. (**B-ii**) Representative plot of tumor preparations stained with anti-human Fas. Percentage of Fas+ cells shown in the top right. (**B-iii**) There was no significant difference in the percentage of cells expressing Fas (*p* = 0.52) or the intensity of Fas expression as measured by median fluorescence intensity (MFI) on flow cytometry (*p* = 0.58). The mean ± S.D. of 9 control tumors and 10 treated tumors is plotted.

Like CD8+ T cells, CD49b+ NK cells can be activated to become cytotoxic effector cells capable of killing target tumor cells through expression of the death receptor FasL. Single cell suspensions from tumors were co-stained with anti-murine CD49b, a pan-NK cell marker and anti-murine FasL (Figure 3(A-i) and Figure 3(A-ii)). Untreated tumors contained a low level of infiltrating activated FasL+CD49b+ NK cells (0.34% ± 0.21%) compared with ^177^Lu-DOTATATE treated tumors which contained a significantly increased number of FasL+ CD49b+ NK cells (0.66% ± 0.29%, *p* = 0.0143) (Figure 3(A-iii)). 

### 3.4. ^177^Lu-DOTATATE Treatment does not Increase Expression of Fas Receptor on Tumor Cells

While increased infiltration of FasL+ NK cells represents an increased capacity for NK cell mediated tumor lysis in ^177^Lu-DOTATATE treated mice, an increased Fas expression by target tumor cells represents increased susceptibility to NK and CD8+ T cell FasL mediated cytotoxic killing [16]. Expression of human Fas on engrafted NCI-H727 cells was assessed by flow cytometry (Figure 3(B-i) and Figure 3(B-ii)). The percentage of cells from untreated controls expressing human Fas (21.7% ± 11.5%) was not significantly different from the percentage of cells expressing Fas following treatment with ^177^Lu-DOTATATE (18.8% ± 8.2%, *p* = 0.52) (Figure 3(B-iii)). Similarly the intensity of Fas receptor expression on tumor cells from untreated (82.9 ± 15.2) and ^177^Lu-DOTATATE treated tumors (was not significantly different 79.4 ± 11.9, *p* = 0.58) (Figure 3(B-iii) and Figure 3(B-iv)).

## 4. Discussion

The treatment of cancer is becoming increasingly multimodal. Current non-operative treatment ranges from the traditional therapies that aim to disrupt the subcellular processes of cell cycling of tumor cells to novel ones that aim to disrupt the microvascular blood supply of tumors. The anti-tumor immune response is increasingly being targeted by new therapies. 

Here we show that PRRT is able to induce an anti-tumor immune response in addition to its ability to disrupt tumor cell cycling through delivery of ionizing radiation. While the tumor inoculum itself is able to induce an immune response we feel that as both the control and treated mice received the same inoculum any observed differences should be attributed to differences in ^177^Lu-DOTATATE PRRT treatment.

The nude NMRI mice used in this study are athymic and consequently lack T cells and mature B cells. They do however have an intact innate compartment including dendritic cells (DCs) and natural killer (NK) cells. The adaptive T cell compartment has been linked to anti-tumor immune responses and clearly the NMRI nude model is limited in its ability to directly interrogate T cell mediated anti-tumor responses. Instead we have examined the DC compartment as a proxy for T cell mediated responses as well as the NK cell compartment.

Physiological activation of tumor specific CD8+ CTLs is tightly controlled and dependent upon cross presentation of tumor peptides on major histocompatibility complex (MHC) class I by specialized antigen presenting cells (APCs), such as DCs and macrophages, to cognate CD8+ T cells [17,18,19]. In addition to specific presentation of cognate antigen on the MHC complex, further stimulatory signals are required for the efficient activation of cognate CD8+ T cells. One of these co-stimulatory signals is CD86. The expression of CD86 is both necessary for and correlated to the efficacy of MHC class I restricted activation of anti-tumor CD8+ T cells by APCs [20,21]. Expression of CD86 is also restricted to APCs. Hence we assayed infiltration of CD86+ cells in tumor tissue as a proxy marker of potential anti-tumor CD8+ T cell responses. We have shown that treatment with ^177^Lu-DOTATATE leads to both increased tumor infiltration by CD86+ APCs and higher level of CD86 expression on these APCs. We speculate this could lead to increased priming and activation of CD8+ CTLs in draining lymph nodes and subsequent tumor specific lysis by activated CD8+ CTLs [19]. Indeed it is well recognized that increased DC infiltration of tumor tissues is positively correlated with host survival in human cancers [22]. We further speculate that the increased presence and activation of CD86+ antigen presenting cells may also increase tumor specific CD4+ T cell activation with all the downstream effects such as increased B cell help and anti-tumor antibody production.

While the adaptive anti-tumor CD8+ CTL response has been shown to be important in the control and eradication of tumor tissue [15], the innate anti-tumor response, mediated in part by NK cells, is also implicated [23]. Activated CD8+ CTLs kill tumor cells that express cognate tumor specific epitopes on MHC molecules. Many tumor cells evade the specific CD8+ CTL mediated anti-tumor response by down-regulation of their surface MHC molecules. Unlike CD8+ CTLs, NK cells rely on a conserved innate recognition of absence of MHC on tumor cells and hence comprise a separate and distinct anti-tumor immune response [23]. NK cells express a wide range of death receptor ligands including FasL and TRAIL, which when ligated with cognate receptors on target tumor cells leads to cell death by apoptosis [24]. One of the most potent pathways by which both NK cells and CD8+ CTLs kill tumor cells is the Fas (CD95) and FasL (CD178/CD95L) death receptor signaling pathway [16]. Upon activation by target cells for lysis, by appropriate cytokines or by CD16a ligation, NK cells have the capacity to up-regulate surface expression of FasL. The subsequent ligation of FasL to its cognate death receptor Fas expressed on target tumor cells results in activation of target cell caspase 8 and cell death [25]. Furthermore, FasL has been also shown to be stored in the same vesicles as perforin and granzyme, the other major mediators of NK cytotoxicity and so is a good measure of NK killing capacity [26,27]. Here we have shown that ^177^Lu-DOTATATE treatment leads to increased tumor infiltration by FasL expressing NK cells. This two-pronged adaptive and innate anti-tumor immune response following ^177^Lu-DOTATATE PRRT may reduce tumor evasion and warrants further investigation in an immune competent mouse model.

We have further investigated the susceptibility of tumor cells to death receptor mediated cytolysis by quantifying the expression of human Fas death receptor on xenografted tumor cells after treatment by ^177^Lu-DOTATATE. Radiotherapy has been shown to increase Fas expression on several types of radiosensitive cancer cells [28,29]. In contrast, we found that treatment with ^177^Lu-DOTATATE did not increase Fas expression on tumor cells in our NET model. Therefore, future optimization of PRRT should aim to increase tumor cell Fas expression to potentially enhance tumor susceptibility to FasL mediated immune killing by FasL expressing NK cells and CD8+ CTLs.

## 5. Conclusions

In summary we have observed that treatment with ^177^Lu-DOTATATE PRRT in our human xenograft tumor model of NET results in increased CD86+ APC infiltration and increased expression of CD86 on these cells. We have furthermore observed that treatment with ^177^Lu-DOTATATE PRRT also increases tumor infiltration by FasL expressing NK cells. Further study in an immune competent model of NET is required to fully dissect the effect of ^177^Lu-DOTATATE PRRT treatment on the tumor immune stroma and particularly the adaptive T cell compartments. Sampling of primary human tumors before and after PRRT treatment to interrogate the immune infiltrate should also be undertaken in parallel. Should our findings be borne out, we speculate that delivery of higher but less frequent doses of ^177^Lu-DOTATATE PRRT may stimulate a better anti-tumor immune response than a low dose/high frequency regime in clinical practice.
